# Nicotinamide Mononucleotide Supplementation Alleviates Doxorubicin-Induced Multi-Organ Fibrosis

**DOI:** 10.3390/ijms25105303

**Published:** 2024-05-13

**Authors:** Fei Wen, Anhua Xu, Wenjing Wei, Shenglong Yang, Zhiliang Xi, Yuanlong Ge, Shu Wu, Zhenyu Ju

**Affiliations:** Key Laboratory of Regenerative Medicine of Ministry of Education, Institute of Aging and Regenerative Medicine, Department of Developmental & Regenerative Medicine, College of Life Science and Technology, Jinan University, Guangzhou 510632, China; wenfei@stu2019.jnu.edu.cn (F.W.); geyuanlong@jnu.edu.cn (Y.G.)

**Keywords:** doxorubicin, nicotinamide mononucleotide, NAD^+^, multi-organ injury, fibrosis

## Abstract

Doxorubicin (DOX) is a potent chemotherapeutic agent known for its multi-organ toxicity, especially in the heart, which limits its clinical application. The toxic side effects of DOX, including DNA damage, oxidative stress, mitochondrial dysfunction and cell apoptosis, are intricately linked to the involvement of nicotinamide adenine dinucleotide (NAD^+^). To assess the effectiveness of the NAD^+^ precursor nicotinamide mononucleotide (NMN) in counteracting the multi-organ toxicity of DOX, a mouse model was established through DOX administration, which led to significant reductions in NAD^+^ in tissues with evident injury, including the heart, liver and lungs. NMN treatment alleviated both multi-organ fibrosis and mortality in mice. Mechanistically, tissue fibrosis, macrophage infiltration and DOX-related cellular damage, which are potentially implicated in the development of multi-organ fibrosis, could be attenuated by NAD^+^ restoration. Our findings provide compelling evidence for the benefits of NMN supplementation in mitigating the adverse effects of chemotherapeutic drugs on multiple organs.

## 1. Introduction

Doxorubicin (DOX), first isolated from Streptomyces peucetius [1], is an effective and widely used anti-tumor drug in chemotherapy for various tumors, including hematological malignancies and solid tumors such as leukemia, lymphoma, breast, ovarian and lung cancer. However, its dose-dependent cardiotoxicity limits its clinical application. Cardiotoxicity is the most common toxic effect of DOX due to its dose accumulation [2]. The main mechanisms by which it induces myocardial injury include DNA damage, mitochondrial dysfunction, fibrosis, oxidative stress, the inflammatory response and cell apoptosis [3]. DOX causes DNA double-strand breaks (DSBs) by binding DNA and Top2β in myocardial cells [4]. Deletion of genes involved in DSB repair leads to the accumulation of DNA damage, which may be crucial in DOX-induced cardiotoxicity [3]. More than 90% of the ATP utilized by myocardial cells is derived from mitochondria [5]. DOX can disrupt the electron transport chain and induce various cellular damages including mitochondrial dysfunction in myocardial cells [6]. Excessive production of reactive oxygen species (ROS) during DOX metabolism also contributes to cardiomyopathy by causing biomolecular damage and activating pro-apoptotic pathways [7]. In addition to the heart, DOX accumulation [8], oxidative damage [9], inflammatory cell infiltration [10] and cytoplasmic vacuolization are also observed in the liver and kidneys of mice treated with DOX [11].

Some chemical drugs and natural products have protective effects on DOX-induced damage [12,13]. For example, geraniol ameliorates DOX-induced kidney injury by alleviating oxidative damage, inflammation and apoptosis [14]. Dexamethasone, the only approved drug, can attenuate the myocardial toxicity of DOX by reducing mitochondrial iron levels [15] and ROS contents [16]. However, some side effects caused by dexamethasone can hinder the effectiveness of chemotherapy and increase the risk of secondary malignant tumors, especially acute myeloid leukemia and myelodysplastic syndromes [17]. Discovering and developing promising drugs that can inhibit ROS production, cell apoptosis and the inflammatory response and enhance mitochondrial function may be effective in ameliorating DOX toxicity [18,19].

As a fundamental molecule in cells, NAD^+^ plays key roles in energy metabolism and mitochondrial function and is involved in counteracting DNA damage and oxidative stress [20]. Supplementing the NAD^+^ precursor nicotinamide riboside (NR) may efficiently alleviate DOX-induced cardiomyopathy by promoting DNA repair and mitochondrial homeostasis [21]. Nicotinic acid riboside (NAR) treatment attenuates DOX-induced myocardial injury through the Nrf-2/p62 pathway [22]. In mice livers, cell death caused by oxidative stress is reduced by NAD^+^ therapy [23], and the antioxidant capacity of cells is enhanced [24]. The biosynthesis of NAD^+^ mainly relies on salvage pathways. As an important intermediate, NMN can be directly converted into NAD^+^ by Nicotinamide mononucleotide adenylyl transferases (NMNATs). Thus, supplementing NMN might be more efficient than other NAD^+^ precursors [25]. Although NMN was reported to prevent cardiotoxicity and improve physical activity in mice [26], no studies have focused on its effect on multi-organ injury. Whether multi-organ injury is associated with NAD^+^ levels or can be alleviated by NMN treatment remains unknown. Here, we report that NMN is beneficial to NAD^+^ levels’ restoration and multi-organ injury alleviation. When boosting NAD^+^, cellular damage, fibrosis and macrophage infiltration caused by DOX are relieved, which may contribute to improved survival in mice.

## 2. Results

### 2.1. Doxorubicin Administration Leads to NAD^+^ Levels Decreasing and Multi-Organ Injury in Mice

**Figure 1:**
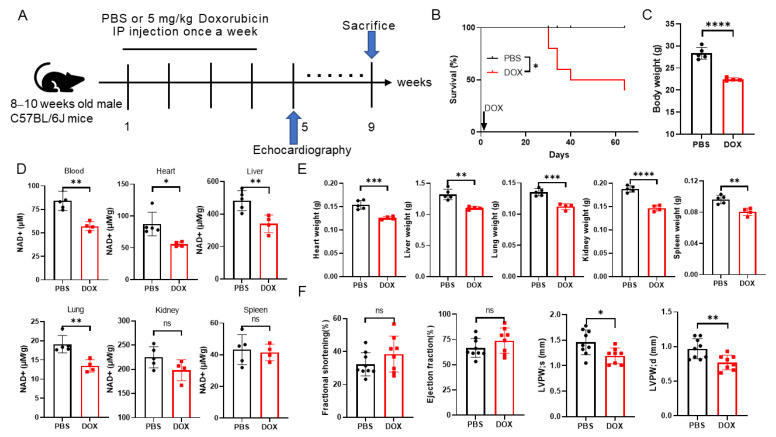
Doxorubicin induces a decline in NAD^+^ levels and multi-organ injury in mice. (**A**) Study design for multi-organ injury in mice. C57BL/6J male mice aged 8–10 weeks were randomly grouped, where PBS or 5 mg/kg doxorubicin was administered via intraperitoneal injection once a week 4 times in the PBS group (n = 10) and DOX group (n = 10), respectively. Echocardiography was performed in week 5 and the mice were sacrificed in week 9. (**B**) Survival curve of mice treated with PBS (n = 10) or doxorubicin (n = 10). (**C**) Body weight changes in week 9 since treatment began. PBS group (n = 5), DOX group (n = 4). (**D**) NAD^+^ contents of whole blood, heart, liver, lungs, kidneys and spleen were measured in week 9. PBS group (n = 5), DOX group (n = 4). (**E**) Weights of different organs harvested from week 9. PBS group (n = 5), DOX group (n = 4). (**F**) Echocardiographic measurements in week 5 since first PBS (n = 9) or DOX injection (n = 8). LVPWs, left ventricular posterior wall thickness in systole; LVPWd, left ventricular posterior wall thickness in diastole. Data are shown as means ± standard deviations. Unpaired 2-tailed Student’s *t*-test was used between two groups (**C**–**F**). Survival rates were measured by log-rank (Mantel−Cox) test (**B**). ns, not significant, * *p* < 0.05, ** *p* < 0.01, *** *p* < 0.001, **** *p* < 0.0001.

Doxorubicin has been reported to induce multi-organ injury including in the heart, liver and lungs [8]. Substantial evidence indicates that NAD^+^ levels play crucial roles in heart and liver injury [22,24,26,27]. In this study, a multi-organ injury mice model was established by a cumulative 20 mg/kg DOX injection. This DOX regimen was chosen based on previous reports in cardiopathy [28] and liver injury models of mice [24]. Echocardiography was performed 1 week after the final DOX treatment, and mice were sacrificed 4 weeks after echocardiographic measurement. The blood and main organs were collected for NAD^+^ levels’ detection (Figure 1A).

To check the relationship between NAD^+^ levels and the organ toxicity of DOX, the NAD^+^ levels in the blood and main organs in mice were detected. The results showed that the NAD^+^ levels of the DOX group in the blood, liver, heart and lungs decreased remarkably but not in the kidneys and spleen (Figure 1D). The survival of the DOX group was 40% that of the PBS group (Figure 1B). Significant reductions were also noted in body weight and organ weights including those of the heart, liver and lungs after DOX injection (Figure 1C,E). Injuries in the main organs were evaluated by Hematoxylin-Eosin (HE) staining, destroyed myocardium architecture, edema in hepatic cells and collapsed alveolar spaces, which indicated the toxicity of DOX to these organs, while no obvious differences were visualized in the kidneys and spleen (Figure S1A). In addition, the heart function and HSPC number were measured. The left ventricular (LV) fractional shortening (FS) and ejection fraction (EF) were comparable in the two groups, and a decreased thickness in the LV posterior wall was observed (Figure 1F), which suggested that there was cardiomyocyte damage although there were no changes in heart function. It was reported that the hematopoietic stem/progenitor cell (HSPC) number was reduced after clinical-dose DOX treatment in mice [29]. Our results showed that the absolute number of HSPCs (LSK [Lineage-, Sca1+, c-Kit+] cells), short-term hematopoietic stem cells (ST-HSCs) and multipotent progenitors (MPPs) were decreased, but no significant changes were detected in long-term hematopoietic stem cells (LT-HSCs) (Figure S1D,E) or the frequencies of T cells, B cells and myeloid cells in the blood after DOX treatment (Figure S1B,C).

These findings indicate that NAD^+^ levels’ decline in the heart, liver and lungs may be closely associated with multi-organ injury and poor survival of mice exposed to DOX. Therefore, whether boosting NAD^+^ by administering NMN can alleviate multiple organ injury is worthy of study.

### 2.2. NMN Supplementation Attenuates Doxorubicin Toxicity to Multiple Organs and Promotes Mice’s Survival by Elevating NAD^+^ Levels

**Figure 2:**
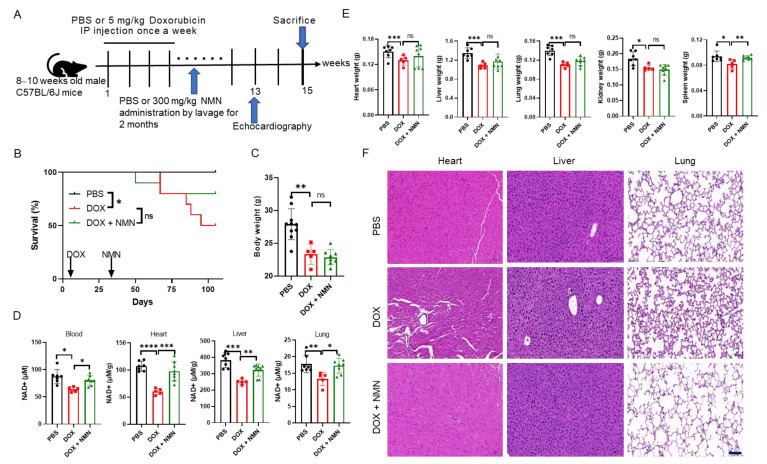
Effects of NMN on NAD^+^ levels and multi-organ injury in doxorubicin-treated mice. (**A**) Experimental description for NMN supplementation in multi-organ-injury mice, where 8–10-week-old male C57BL/6J mice were separated into 3 groups randomly: PBS group (n = 10), DOX group (n = 10), DOX + NMN group (n = 10). PBS or 5 mg/kg doxorubicin was intraperitoneally injected for the PBS, DOX or DOX + NMN group, respectively, once a week 4 times. Then, PBS or 300 mg/kg NMN was administered for 8 weeks. Echocardiographic analysis was performed in week 13 and mice were sacrificed in week 15. (**B**) Survival rates of different groups in week 15 (n = 10 mice per group). (**C**) Body weight changes in week 15 (n = 5–10). (**D**) NAD^+^ levels in blood and organs after NMN supplementation (n = 5–8). (**E**) Weights of different organs at the day of sacrifice (n = 5–10). (**F**) Representative HE staining of heart, liver, lungs, kidneys and spleen harvested from week 15. Scale bar, 100 μm. Data are shown as means ± standard deviations. Differences of three groups were assessed by one-way ANOVA test (**C**–**E**). Survival rates were measured by log-rank (Mantel−Cox) test (**B**). ns, not significant, * *p* < 0.05, ** *p* < 0.01, *** *p* < 0.001, **** *p* < 0.0001.

To investigate the potential of NAD^+^ replenishment in mitigating the toxicity of DOX across various organs in mice, an analysis was conducted of the NAD^+^ levels and morphological alterations in the heart, liver and lungs following 2-month NMN administration (Figure 2A). As the results showed, decreased NAD^+^ levels in the blood and organs were restored after NMN treatment (Figure 2D). Although the body weight and weights of multiple organs were similar when compared to the DOX group (Figure 2C,E), the survival rate of the DOX + NMN group was improved (Figure 2B). As visualized by HE staining, the destroyed myocardium architecture, edema in hepatocytes and collapsed alveolar spaces were almost completely reversed (Figure 2F). Additionally, the FS and EF of the DOX + NMN group were comparable to those of the PBS group, indicating the benefits of NMN for cardiac function recovery (Figure S2A,B). No evident changes were found in the absolute numbers of LSKs, LT-HSCs, ST-HSCs and MPPs in the bone marrow (Figure S2E,F) or the frequencies of B cells and myeloid cells in the blood (Figure S2C,D) after NMN administration. These observations imply that NAD^+^ replenishment may attenuate multi-organ injury and decrease mortality induced by DOX.

### 2.3. NMN Supplementation Alleviates Fibrosis of Heart, Liver and Lungs in Mice

**Figure 3:**
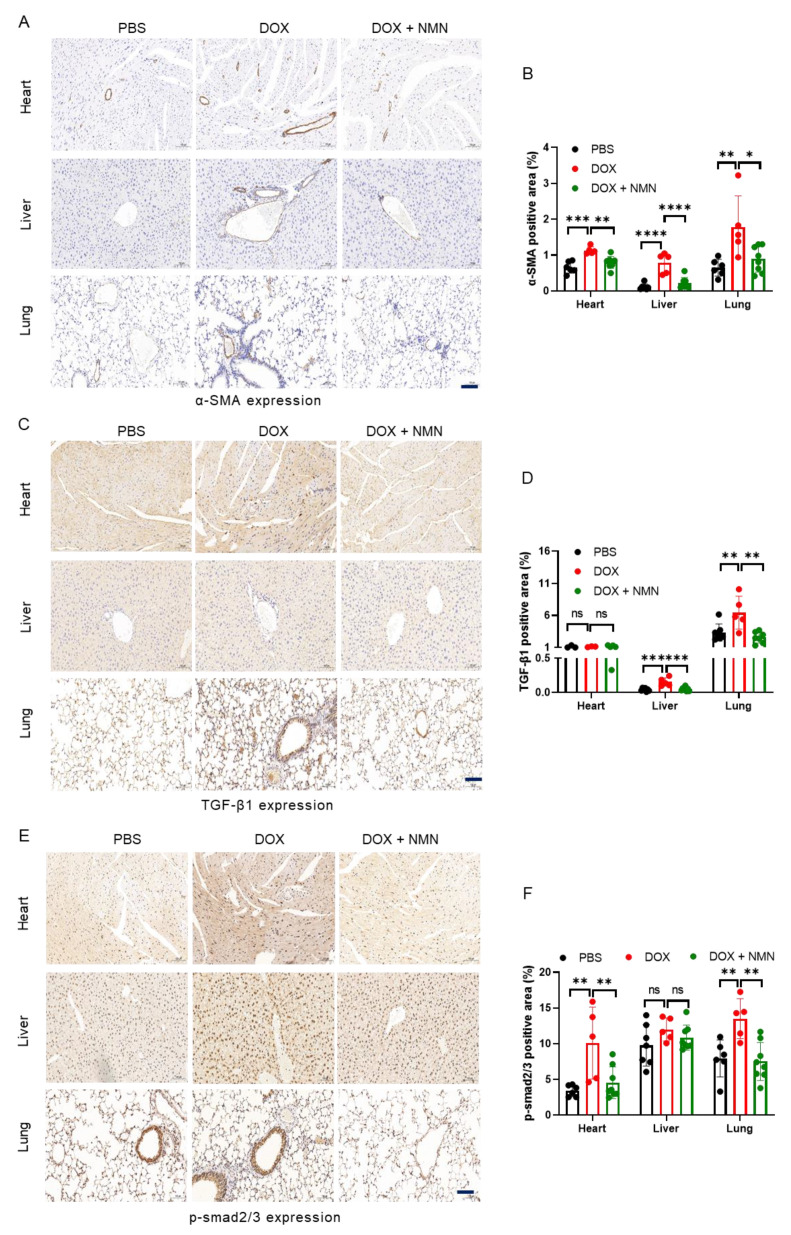
Effects of NMN on fibrosis in mice exposed to doxorubicin. (**A**,**C**,**E**) Representative immunohistochemistry results for α-SMA (**A**), TGF-β1 (**C**) and p-smad2/3 (**E**) in heart, liver and lungs harvested from week 15. (**B**,**D**,**F**) Bar graphs show α-SMA (**B**), TGF-β1 (**D**) and p-smad2/3 (**F**) positive areas (n = 5–8). Scale bar, 100 μm. Data are shown as means ± standard deviations. Differences of three groups were assessed by one-way ANOVA test (**B**,**D**,**F**). ns, not significant, * *p* < 0.05, ** *p* < 0.01, *** *p* < 0.001, **** *p* < 0.0001.

Tissue fibrosis was reported in mice exposed to DOX [21,30], and epithelial-to-mesenchymal transition (EMT) along with endothelial-to-mesenchymal transition (EndoMT) are crucial in excessive myofibroblast generation and progression of fibrosis. The mesenchymal marker alpha-smooth muscle actin (α-SMA) was activated in resident fibroblasts during cardiac fibrosis [31]. Thus, α-SMA was detected in the heart, liver and lungs by immunohistochemistry (IHC). The increased expression of α-SMA in the DOX group was reduced by NMN supplementation (Figure 3A,B). Transforming growth factor-β1 (TGF-β1), which is commonly elevated in patients with cardiomyopathy [32], stimulates collagen synthesis, fibroblast proliferation and cell transformation [33]. This factor is always detected in plasma after chemotherapy due to its association with the risk of posttreatment complications developing [34]. Here, TGF-β1 expression of the DOX group was higher in the mice liver and lungs, and it was mitigated by NMN treatment (Figure 3C,D). Similarly, mothers against decapentaplegic homolog 2/3 (p-smad2/3), downstream of TGF-β1, was suppressed in the heart and lungs by boosting NAD^+^ (Figure 3E,F). These findings suggest that NMN administration can alleviate tissue fibrosis in mice.

### 2.4. NMN Treatment Reduces Macrophage Infiltration in Mice

**Figure 4:**
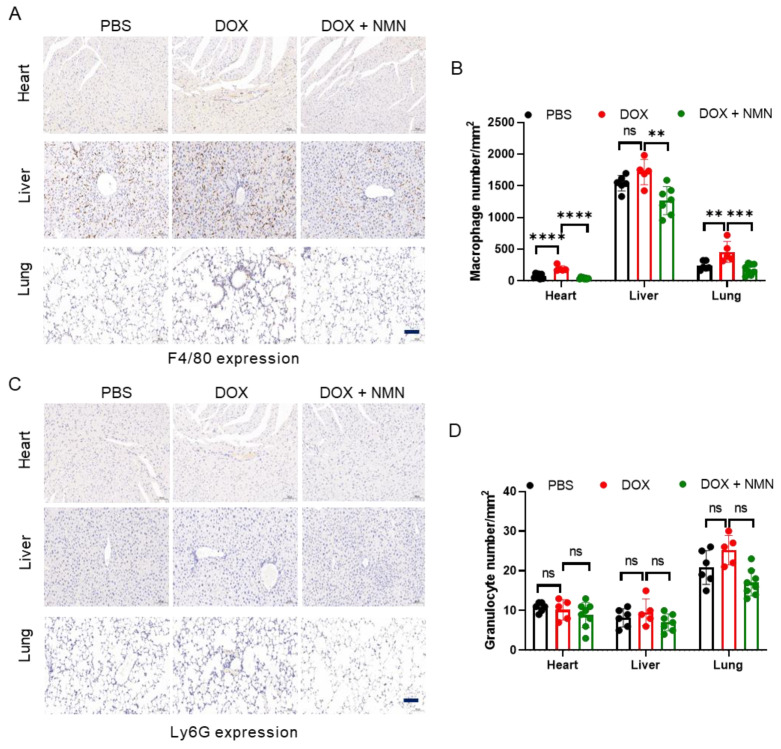
Macrophage and granulocyte infiltration in tissues following NAD^+^ replenishment. (**A**,**C**) Representative immunohistochemistry results for F4/80 (**A**) and Ly6G (**C**) in heart, liver and lungs harvested from week 15. (**B**,**D**) Bar graphs show F4/80 (**B**) and Ly6G (**D**) positive numbers (n = 5–8). Scale bar, 100 μm. Data are shown as means ± standard deviations. Differences of three groups were assessed by one-way ANOVA test (**B**,**D**). ns, not significant, ** *p* < 0.01, *** *p* < 0.001, **** *p* < 0.0001.

When tissues are damaged by toxic stimuli, immune cells are recruited, releasing various cytokines and chemokines, triggering inflammatory reactions and activating fibroblasts. Immune cells play regulatory roles in the progression of fibrosis. Therefore, we detected macrophage and granulocyte infiltration in the mice heart, liver and lungs through IHC staining with F4/80 and Ly6G, respectively. The results indicated that NMN treatment reduced macrophage infiltration (Figure 4A,B) but did not affect granulocytes (Figure 4C,D). CD38(+) macrophages caused more NAD^+^ consumption, which may aggravate tissue damage, in the DOX group. These results imply that supplementing NMN could reduce macrophage infiltration and be beneficial to NAD^+^ levels’ restoration.

### 2.5. Boosting NAD^+^ Reduces Cellular Damage and Suppresses EMT Inducers’ Expression

**Figure 5:**
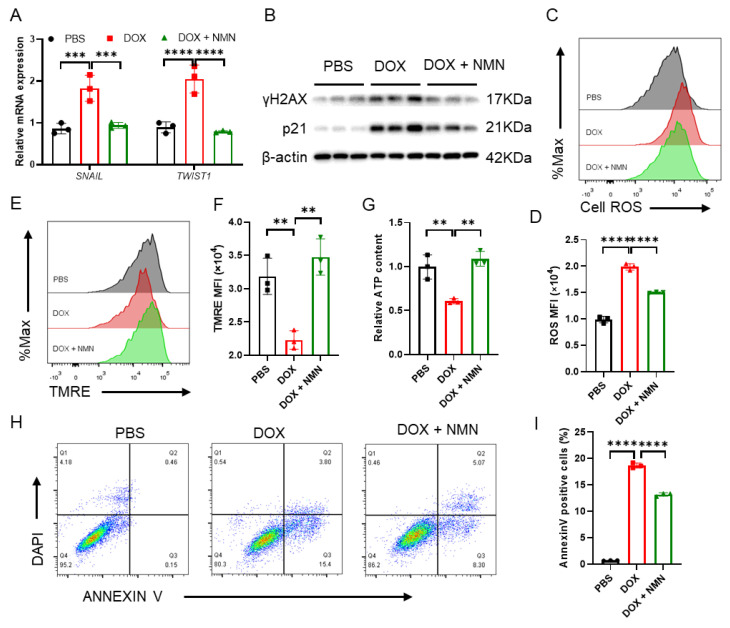
Boosting NAD^+^ attenuates doxorubicin-induced cellular damage in fibroblast MRC5 cells. (**A**) Relative mRNA expression of *SNAIL* and *TWIST1* after treatment in human fibroblast MRC5 cells (n = 3 per group), where β-actin is used as an endogenous control. (**B**) Western blot of p21 and γH2AX in MRC5 cells treated with PBS, DOX or DOX + NMN (n = 3 per group), where β-actin is used as a loading control. (**C**) Cell ROS measurements of PBS-, DOX- or DOX + NMN-treated MRC5 cells by FACS (n = 3 per group). (**D**) Quantitative analysis of the mean fluorescence intensity of cell ROS (n = 3). (**E**) Mitochondrial membrane potential tested by FACS (n = 3 per group). (**F**) Quantitative analysis of the mean fluorescence intensity of mitochondrial membrane potential (n = 3 per group). (**G**) Cellular ATP content in MRC5 cells (n = 3 per group). (**H**,**I**) Dot plots of annexin V-FITC and 4,6-diamidino-2-phenylindole (DAPI) staining (**H**) and the percentages of annexin V-positive cells (**I**) (n = 3 per group). Data are shown as means ± standard deviations. Differences of three groups were assessed by one-way ANOVA test (**A**,**D**,**F**,**G**,**I**). ns, not significant, ** *p* < 0.01, *** *p* < 0.001, **** *p* < 0.0001.

The primary mechanisms of DOX toxicity to cells include DNA damage, oxidative stress, mitochondrial dysfunction and apoptosis. DNA damage caused by ionizing radiation, ultraviolet light or DNA toxic drugs leads to rapid phosphorylation of H2A.X at Ser139 and labeling of damaged DNA regions with the γH2AX protein [35]. DOX causes p53 pathway transcriptomic changes, which can be regulated by NMN [26]. Activation of p53-p21 can result in cell cycle arrest and a DNA damage response [36]. Here, we found that NMN supplementation could reduce γH2AX and p21 expression in MRC5 fibroblast cells exposed to DOX (Figure 5B) as well as ROS levels (Figure 5C,D). These findings indicate that NMN treatment may help alleviate DNA damage and oxidative stress induced by DOX. Additionally, NMN was indicated to enhance mitochondrial function by promoting mitochondrial transmembrane potential (MTP) and ATP contents (Figure 5E–G), counteracting cell apoptosis (Figure 5H,I). Furthermore, as key regulators in EMT, which is crucial for fibrosis progression [37], the mRNA expression of SNAIL and TWIST1 was suppressed by NMN treatment (Figure 5A). These results suggest that the cell toxicity of DOX and pro-fibrosis factors’ expression could be reduced by NMN treatment.

## 3. Discussion

DOX is believed to exert its anti-cancer effect by inhibiting cell proliferation and blocking the cellular DNA replication process. However, it has severe adverse effects, especially cardiac toxicity including left ventricular dysfunction, progressive left ventricular remodeling and heart failure. Even low-dose DOX may lead to cardiomyopathy [38], which can be detected several days, months or even years after treatment [39]. Additionally, DOX can be accumulated in the heart, liver and lungs [8], causing fibrosis [4,24] and bone marrow suppression [29]. In DOX-treated mice, NAD^+^ boosting can protect from cardiomyopathy, but whether multi-organ injury caused by DOX is closely associated with NAD^+^ levels or whether it can be reversed by NMN administration remains elusive. In this study, the two major findings were as follows: First, DOX causes various organs to become injured, including the heart, liver and lungs, accompanied by NAD^+^ levels’ decline. Second, multi-organ fibrosis, macrophage infiltration, and cellular damage can be improved by NMN administration.

The toxicity of DOX depends on its cumulative dose; different regimens of DOX were reported to cause body weight loss and mortality in animals (30–52%) [26,40,41], and accumulation of 20–25 mg/kg DOX was closely correlated with cardiac malfunction in mice [27,42,43,44]. Consistently in this study, a high mortality rate (60%) and reduced body weight (~20%) and organ weights were observed. Cardiac dysfunction was not evident, although there was a decrease in the thickness of the left ventricular posterior wall, which may have been due to the survivor effect and different time points. In previous research, no significant changes were found in EF or FS at 53 and 30 days [26] or EF at 5 weeks following DOX treatment [45]. Most studies on DOX toxicity focused on individual organs [10,14,27,46], and NAD^+^ supplementation was suggested as an effective detoxification approach. Here, multi-organ injury and NAD^+^ levels were detected in mice exposed to DOX. Our results imply links between insufficient NAD^+^ and organ injury in the heart, liver and lungs. In the bone marrow, a clinical dose of DOX can reduce the number of HSPCs in mice [29], and similarly, in this study, we observed declines in LSK, ST-HSCs and MPPs, indicating the toxic effects of DOX on hematopoietic cells. These toxic side effects of DOX resulted in the poor survival of mice, and the decrease in NAD^+^ levels in the injured organs suggested that replenishing NAD^+^ may potentially counteract the multi-organ toxicity of DOX.

NAD^+^ precursors’ supplementation is an appealing approach to mitigating the toxicity of DOX [21,22,26]. For instance, intravenous administration of NR can promote physical activity by enhancing cardiac function and inhibiting fibrous tissue formation [21]. Promoting NAD^+^ through NMN supplementation works to counteract weight loss and cardiac dysfunction by mitigating mitochondrial dysfunction, oxidative stress, cell apoptosis and inflammation, which may contribute to improving mice survival [26]. It has been documented that various biomolecules, including sirtuins, poly(ADP-ribose) polymerases (PARPs), CD38, CD157 and sterile alpha and TIR motif containing 1 (SARM1), are known to consume NAD^+^, while nicotinamide phosphoribosyltransferase (NAMPT), NR kinases (NRK), NMNAT1/2/3, nicotinamide adenine dinucleotide synthase (NADS) and quinolinate phosphoribosyltransferase (QPRT) are involved in the synthesis of NAD^+^ through de novo, Preiss–Handler and salvage pathways [47,48]. However, specific molecular markers associated with alterations in NAD^+^ levels within organs remain poorly defined, and the measurement of NAD^+^ levels is typically accepted when administering NAD^+^ precursors [22,26,49,50]. Hence, we measured the NAD^+^ levels of the blood and major organs after NMN treatment and a significant increase in NAD^+^ levels was observed in blood, heart, liver and lungs of the DOX + NMN group. Moreover, histological analysis indicated that NAD^+^ levels’ restoration through NMN administration can attenuate the toxicity of DOX in these organs. Thus, the DOX + NMN group exhibited a higher survival, although there were no changes in body weight and organ weight. In addition, no evident changes were observed in cardiac function and numbers of HSPCs. The powerful hematopoietic reconstitution capacity of HSPCs and the survivor effect may be responsible for the lack of difference observed in these instances, as well as the distinct administration strategies of DOX and NMN. As DOX and NAR cotreatment could restore heart function on day 15 since DOX treatment began [22], preventative NMN supplementation may be a better regimen, which may attenuate body weight loss and cardiotoxicity and improve physical function [26]. Moreover, NMN supplementation may promote the mitochondrial oxidative function, enhance energy metabolism and reduce adipogenesis [51], which could be among the reasons for no evident rescues in body weight and organ weight loss after NMN treatment. Fibrosis induced by DOX play key roles in cardiopathy development [21,30,43]. Our results suggested that NMN administration not only suppresses α-SMA, TGF-β1 and p-smad2/3 expression, which reflects relieved fibrosis, but also reduces the infiltration of macrophages in the heart, liver and lungs. It is reported that anthracycline induces macrophage phenotype transition [52], and that CD38(+) macrophages can accelerate NAD^+^ consumption [53]. Decreased macrophage infiltration may contribute to NAD^+^ levels’ restoration. Therefore, NMN administration may prevent tissue fibrosis, which could be beneficial to the function of multiple organs and mice’s survival.

In terms of its mechanism, DOX causes dysregulation of p53 target genes, including those for the cell cycle, DNA damage, apoptosis and genetic changes related to mitochondrial function, ROS and inflammation. NMN supplementation restores all these genes to basal levels [26]. DOX not only directly affects DNA replication but also produces ROS during the process of DOX reduction to 7-deoxyridamycin [54]. Excessive ROS accumulation leads to oxidative stress, mitochondrial dysfunction and macromolecular damage including to DNA, RNA and proteins [55]. In mice, elevating NAD^+^ levels can rescue GSH decline in the liver and reduce astrocyte and neuronal death caused by oxidative stress, as well as mitochondrial permeability transition [23,56]. The protective effect of NAD^+^ precursors NMN, NR and NAR on DOX-induced cardiotoxicity may be mediated by the activation of SIRT1 [57], which can prevent oxidative damage [58], restore mitochondrial biogenesis and inhibit cell apoptosis [59]. NMN is involved in mitochondrial biogenesis and NAD^+^ metabolism, reducing the overexpression of genes related to DNA damage and cell apoptosis, such as p21 in the heart [26]. Due to the crucial role of NAD^+^ in energy metabolism in eukaryotic cells, NAD^+^ homeostasis is essential for stable mitochondrial function and ATP production [60]. In this study, DNA damage accompanied by increased ROS, mitochondrial dysfunction and cell apoptosis in MRC5 cells exposed to DOX was relieved by NMN treatment. SNAIL and TWIST1 are two crucial EMT inducers that can enhance EMT by inhibiting epithelial and elevating stromal marker expression [37]. Furthermore, they participate in the regulation of collagen synthesis and immune cells’ infiltration, thereby promoting fibrosis [37]. In fibroblasts, we found that mRNA levels of SNAIL and TWIST1 were suppressed by NMN supplementation. Cell cycle arrest during fibrotic injury is a functional consequence of the EMT program [37], and macrophages’ recruiting for immunosurveillance under stress is p21 dependent [61]. Suppressed p21 expression implies that cell stress is relieved, thus leading to a reduction in macrophage infiltration in tissues. The TGF-β/p-smad3 pathway plays pivotal roles in cardiac damage induced by DOX [28], and tissue-resident macrophages secrete pro-inflammatory and fibrotic factors, such as TGF-β1, which can promote the activation of stromal cells, synthesis of collagen and release of cytokines like TNF-α and IL-1 in chronic inflammatory conditions, which contribute to EMT [62,63]. In vivo, we found that supplementing NMN suppresses the TGF-β/p-smad3 pathway and reduces macrophage infiltration. Additionally, chronic inflammation impairs the clearance function of macrophages, making it difficult to remove excessive collagen in a timely fashion, which may accelerate fibrosis progression.

In conclusion, NAD^+^ levels’ decrease is linked to weight loss and multiple organs’ injury, leading to mortality in mice treated with DOX. Boosting NAD^+^ through NMN supplementation reduces cellular damage, tissue fibrosis and macrophage infiltration. All these benefits contribute to alleviating multi-organ fibrosis and promoting the survival of mice. Optimizing the dosage and strategies of NMN treatment, such as supplementing NMN before DOX treatment, may thus be effective in ameliorating the toxic effects of chemotherapy.

## 4. Materials and Methods

### 4.1. Animal Studies

Male C57BL/6J mice were purchased from gempharmatech at 8–10 weeks of age. A standard chow diet and clean water were provided ad libitum. Animals were housed (5 mice per cage) on a 12 h light–dark cycle at 22 ± 2 °C and 55 ± 10% relative humidity. After one week’s acclimating, the mice were randomly divided into three groups as follows: a PBS group (n = 10), a DOX group (n = 10) and a DOX + NMN group (n = 10). Mice in the DOX and DOX + NMN groups were intraperitoneally injected with DOX (5 mg/kg) once weekly for 4 weeks. Then, mice of the DOX + NMN group were given NMN (300 mg/kg) every day for 8 weeks via intragastric administration. Death events were checked and recorded every day, and the body weight was measured once a week.

Freshly isolated tissues including the heart, liver, lungs, kidneys and spleen were fixed in 10% paraformaldehyde and embedded in paraffin for histological evaluation. For NAD^+^ extraction and measurement, 10–20 μg tissues was mixed with 1 mL 10% (*w*/*v*) trichloroacetic acid or snap-frozen in liquid nitrogen and transferred to a −80 °C refrigerator.

### 4.2. Echocardiography

The animals were anesthetized with a mixture of 2% isoflurane–98% oxygen in an anesthesia machine before measurement. Echocardiographic images were recorded with a heart rate at about 400 beats/minute by a VINNO 6 LAB (Vinno Inc., Suzhou, China). Cardiac function parameter analysis was performed in the M mode. Ejection fractions, fractional shortening, posterior wall thickness at diastole (LVPWd) and posterior wall thickness at systole (LVPWs) were measured from the right parasternal short axis.

### 4.3. Histology

Freshly isolated tissues were fixed in 10% paraformaldehyde and embedded in paraffin for histological evaluation. Then, 5 µm sections were cut and stained with hematoxylin and eosin. Representative images were acquired with a scanner and Slide viewer Software (3D histech, version 2.5.0.143918), and 20× visual fields were selected for each slide.

### 4.4. Immunohistochemical Analysis

Tissues were fixed in 10% paraformaldehyde and embedded in paraffin, 5 µm sections of the heart, liver and lungs were deparaffinized and then they were dehydrated in an ascending series of ethanol and antigen retrieval. Next, sections were blocked with 3% H_2_O_2_ in methanol for 15 min to inactivate endogenous peroxidases. After antigen repair, the slides were cooled naturally, placed in PBS and washed on a shaker 3 times, with 5 min per time. The slides were blocked with 5% (*v*/*v*) rabbit serum and 3% (*w*/*v*) BSA and then incubated overnight at 4 °C with one of the following primary antibodies: α-SMA (Rabbit Anti-α-SMA, 1:1000; Solarbio, Beijing, China), TGF-β1 (Rabbit Anti-TGF-β1 Polyclonal antibody, 1:400; Proteintech, Wuhan, China), p-SMAD2/3 (Rabbit Anti-Phospho-Smad2-S465/467+Smad3-S423/425, 1:100; Solarbio, Beijing, China), F4/80 (Rabbit Anti-F4/80 Polyclonal antibody, 1:5000; Proteintech, Wuhan, China) or Ly6G (Rat Anti-Ly6G Polyclonal antibody, 1:400; Proteintech, Wuhan, China). After washing in PBS 3 times, for 5 min per time, the slices were slightly dried and stained with HRP-labeled secondary antibody at room temperature for 50 min. Then, the slides were stained with hematoxylin following washing in PBS 3 times, for 5 min per time. The sections were stained in DAB solution and images were acquired with a scanner and Slide viewer Software (3D histech, version 2.5.0.143918), with 20× visual fields selected for each slide.

### 4.5. NAD^+^ Extraction and Measurement

For NAD^+^ extraction, 100 μL blood samples were acquired in anticoagulant EDTA or 10–20 μg organ samples were homogenized (70 Hz, run for 15 s and paused for 10 s, 10 times) using a homogenizer in 10% (*w*/*v*) trichloroacetic acid for complete lysis, then centrifuged at 4 °C 12,000× *g* for 15 min. The top aqueous layer was transferred to new tubes and the NAD^+^ extraction buffer (1,1,2-trichloro-1,2,2-trifluoroethane: trioctylamine = 3:1) was added at a ratio of 1:2, then this was mixed by vortexing and centrifuged at 4 °C 200× *g* for 1 min. The top aqueous layer containing NAD^+^ was transferred to new tubes and we added 1 M Tris to adjust the pH to 8.0. For NAD^+^ levels’ measurement, 100 μL NAD^+^ containing supernatants was mixed in a 96-well plate at room temperature with 100 μL 61 mM glycylglycine buffer (pH 7.4) containing 10% CCK-8, 13 units/mL alcohol dehydrogenase, 100 mM nicotinamide and 5.7% ethanol. Samples were measured at A450 nm by a microplate reader (Biotek, Burlington, VT, USA) after 30 min incubation at 37 °C, and the results were calibrated with a NAD^+^ standard curve.

### 4.6. Flow Cytometry

The cells were analyzed by a LSRFortessa cell analyzer (BD Bioscience, Franklin Lakes, NJ, USA). In this step, 100 μL blood samples were collected in anticoagulant EDTA and 10 μL blood was used for FACS analysis. After red blood cells’ lysis, they were resuspended in staining medium (0.5% BSA in PBS) and incubated with antibodies against CD4 (RM4-5, Biolegend, San Diego, CA, USA), CD8 (53-6.7, Biolegend, San Diego, CA, USA), CD11b (M1/70, Biolegend, San Diego, CA, USA) and B220 (RA3-6B2, Biolegend, San Diego, CA, USA) at 4 °C for 30 min. Bone marrow cells isolated from mice hind legs were incubated in a lineage cocktail containing antibodies against CD4 (RM4-5, Biolegend, San Diego, CA, USA), CD8 (53-6.7, Biolegend, San Diego, CA, USA), Ter-119 (TER-119, Biolegend, San Diego, CA, USA), CD11b (M1/70, Biolegend, San Diego, CA, USA), Gr-1 (RB6-8C5, Biolegend, San Diego, CA, USA) and B220 (RA3-6B2, Biolegend, San Diego, CA, USA) at 4 °C for 30 min. After washing with PBS, the cells were resuspended in staining medium and incubated with antibodies against CD34 (RAM34, BD Bioscience, Franklin Lakes, NJ, USA), CD48 (HM48-1, Biolegend, San Diego, CA, USA), IL-7R (A7R34, Invitrogen, Waltham, MA, USA), Flt3 (A2F10, Invitrogen, Waltham, MA, USA), CD150 (TC15-12F12.2, Biolegend, San Diego, CA, USA), Sca1 (E13-161.7, Biolegend, San Diego, CA, USA), c-Kit (ACK2, Invitrogen, Waltham, MA, USA), CD16/32 (93, Invitrogen, Waltham, MA, USA) and streptavidin (Invitrogen, Waltham, MA, USA) at 4 °C for 2 h. DAPI (1 μg/mL) was used for dead cells’ staining.

### 4.7. Cell Culture and Drug Treatment

MRC5 cells were seeded at 2 × 10^5^/well in a 12-well plate 12 h before the experiment, and then the cells were incubated in DMEM (10% FBS, 1% P/S) containing 1 μM DOX or PBS for 3 h. Later, the medium within DOX was abandoned and the cells were washed in 1 mL PBS and cultured in medium with or without 20 μM NMN for 48 h.

### 4.8. RNA Purification and Real-Time PCR

Freshly isolated cells were used for the total RNA extraction with Trizol (Takara, Osaka, Japan) reagents. Complementary DNA was then synthesized from 1 μg RNA using a First-Strand cDNA Synthesis kit according to the manufacturer’s instructions (Vazyme, Nanjing, China). The RT-PCR analysis was performed in the Real-Time System (Thermo fisher, Waltham, MA, USA) and SYBR Green Mix. The relative expression levels of genes of interest were calculated by the relative delta-delta-Ct method. The expression of β-actin was used as the internal control. Genes and primer sequences were as follows: β-actin F 5′-TTGCCGACAGGATGCAGAA-3′, β-actin R 5′-GCCGA-TCCACACGGAGTACT-3′; TWIST1 F 5′-CTCAAGAGGTCGTGCCAATC-3′, TWIST1 R 5′-CCCAGTATTTTTATTTCTAAAGGTGTT-3′; SNAIL F 5′-GGCAATTTAACAATGTCTGAAAAGG-3′, SNAIL R 5′-GAATAGTTCTGGGAGACACATCG-3′.

### 4.9. Analysis of the Cell Apoptosis

An Annexin V Apoptosis Detection Kit (BD Pharmingen, Franklin Lakes, NJ, USA) was used for the apoptosis analysis according to the instructions. MRC5 cells were treated with PBS, 2 μM DOX or 2 μM DOX + 20 μM NMN in medium (10% FBS, 1% P/S) for 12 h. Then cells were collected and incubated with FITC-Annexin V antibody for 15 min at room temperature, and DAPI (1 μg/mL) was used for nuclear staining.

### 4.10. Western Blot Analysis

Cells were denatured at 100 °C for 5 min in Laemmli buffer and separated in 12% SDS-PAGE followed by incubation with primary antibodies against γH2AX (1:2000, CST, Boston, MA, USA), p21 (1:2000, Abcam, Cambridge, UK) and β-actin (1:50,000, Abclonal, Wuhan, China) at 4 °C overnight. After washing with TBST 3 times, they were incubated with HRP-conjugated secondary antibodies (1:2000, CST, Boston, MA, USA) for 1 h at room temperature.

### 4.11. Cell ROS and Mitochondrial Transmembrane Potential Detection

Cells were collected and washed with 1 mL PBS, after centrifuging and discarding the supernatant, the cells were incubated with a DCFH-DA (1:1000 *v*/*v*, Beyotime, Beijing, China) or TMRE (1:1000 *v*/*v*, Beyotime, Beijing, China) probe in a medium without FBS at 37 °C for 30 min. Then, the cells were washed 3 times in medium without FBS, and we detected cell ROS or MTP with FACS, respectively.

### 4.12. ATP Content

Cellular ATP concentrations were detected with an ATP detection kit (Beyotime, Beijing, China). MRC5 cells were lysed according to the manufacturer’s instructions. Cells in 6-well plates were lysed in 200 μL lysis buffer and centrifuged at 4 °C and 12,000× *g* for 5 min; then, 20 μL supernatant was used for ATP measurement. ATP contents were measured by a microplate reader (Biotek, Burlington, VT, USA) and standardized to nmol/mg protein, and relative ATP contents were given.

### 4.13. Statistics

Data were analyzed using GraphPad Prism (Version 9.0.0, San Diego, CA, USA) and are shown as means ± standard deviations. An unpaired 2-tailed Student’s *t*-test was used to compare two experimental groups. Differences in three groups were assessed by a one-way ANOVA test. Variance in survival rates was measured by a log-rank (Mantel−Cox) test. ns, not significant, * *p* < 0.05, ** *p* < 0.01, *** *p* < 0.001, **** *p* < 0.0001.

## Data Availability

Data are contained within this article.

## Supplementary Materials

The following supporting information can be downloaded at https://www.mdpi.com/article/10.3390/ijms25105303/s1.
