# Supplementary material for: Nicotinamide Mononucleotide Supplementation Alleviates Doxorubicin-Induced Multi-Organ Fibrosis
Source: Int J Mol Sci. 2024 May 13;25(10):5303. doi: 10.3390/ijms25105303 (PMC11120852; doi:10.3390/ijms25105303)
Supplement: Supplementary file 1 [file ijms-25-05303-s001.zip › ijms-2989017-supplementary.pdf]

Figure S1, Related to Figure 1.

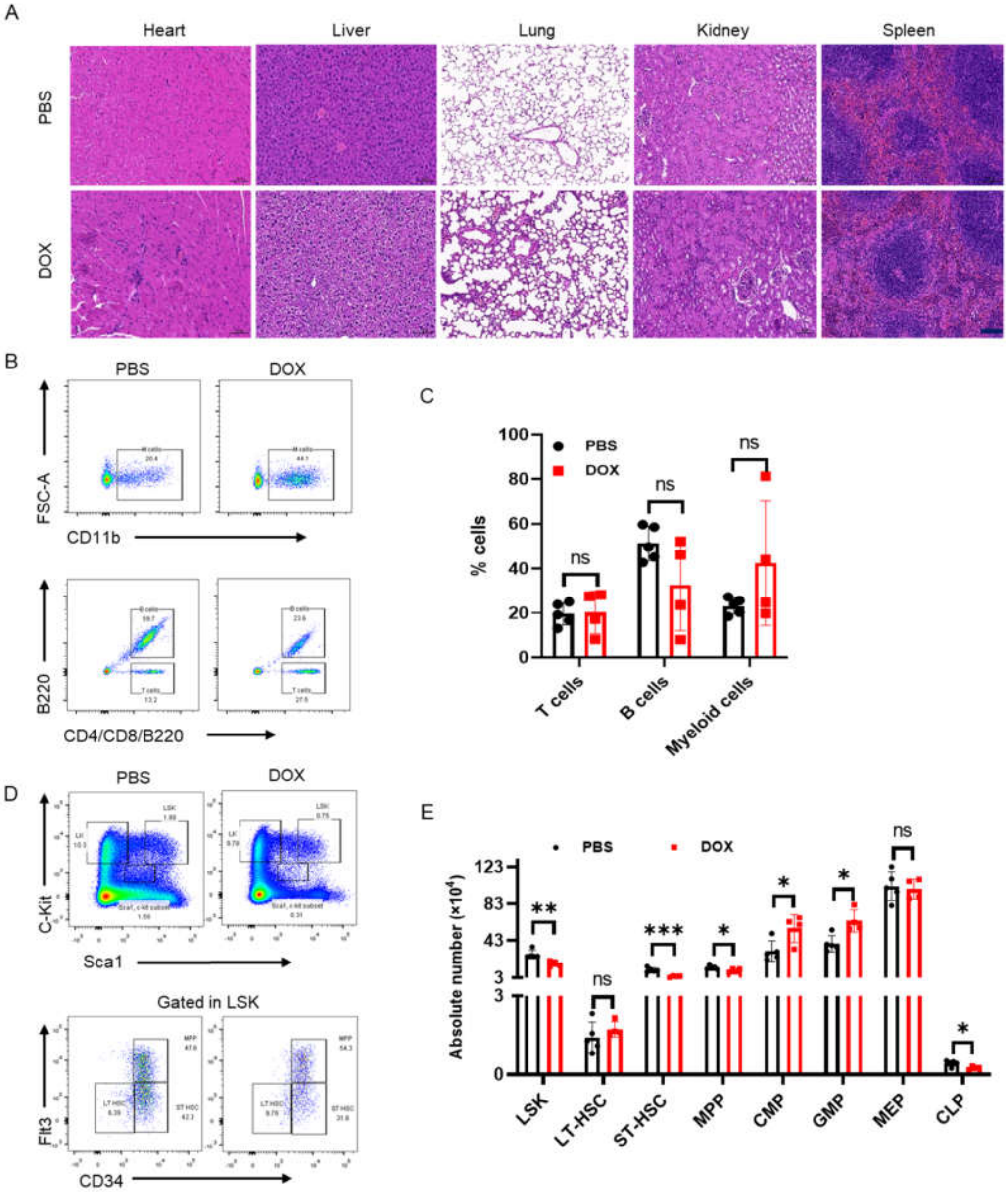

Figure S1. Toxicity of doxorubicin to multiple organs and HSPCs. (**A**) Representative HE staining of heart, liver, lungs, kidneys and spleen. Scale bar, 100μm. (**B**) Representative FACS profiles are shown of peripheral blood (PB) cells in week 9. (**C**) Percentages of myeloid (CD11b+), B (B220+) and T (CD4+/CD8+) cells in PB samples from PBS (n = 5) and DOX (n = 4) group mice. (**D**) FACS analysis of LSK, LT-HSC, ST-HSC and MPP cells in bone marrow. Representative FACS dot plots are shown. (**E**) Numbers of LSKs, LT-HSCs, ST-HSCs, MPPs, GMPs, MEPs, CMPs and CLPs in bone marrow from PBS (n = 5) and DOX (n = 4) group mice. Data are shown as means ± standard deviations. Unpaired 2-tailed Student's t-test was used between two groups (**C**,**E**). ns, not significant, *p<0.05, **p<0.01, ***p<0.001.

Figure S2, Related to Figure 2.

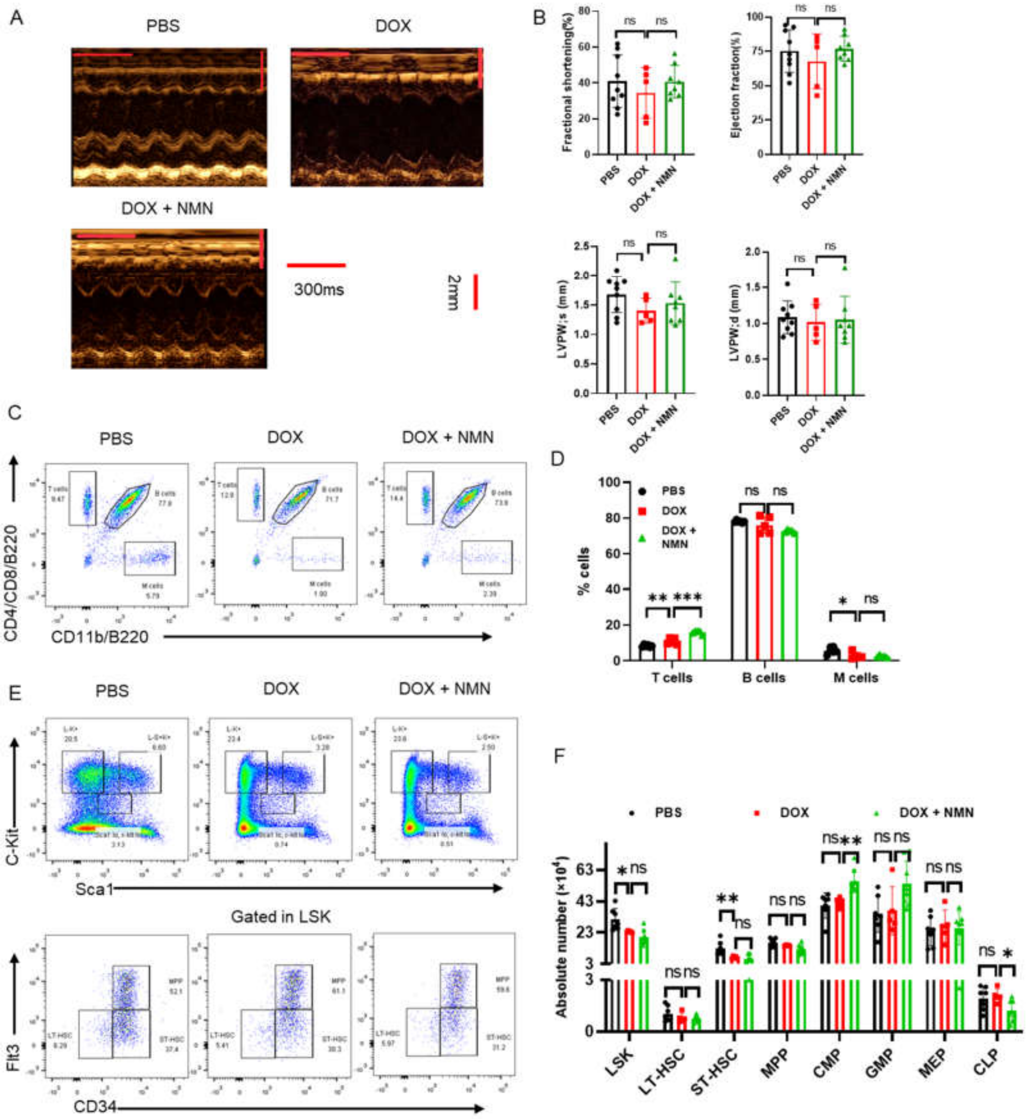

Figure S2. Effects of NMN administration on heart function and HSPC numbers. (**A**) Representative M-mode echocardiograms in week 13. (**B**) Echocardiographic measurement for fractional shortening, ejection fraction, LVPWs and LVPWd (n = 5–8). LVPWs, left ventricular posterior wall thickness in systole; LVPWd, left ventricular posterior wall thickness in diastole. (**C**) Representative FACS profiles are shown of PB cells in week 15. (**D**) Percentages of myeloid (CD11b+), B (B220+), and T (CD4+/CD8+) cells in PB samples from PBS (n = 7), DOX (n = 5) and DOX + NMN (n = 8) group mice. (**E**) Representative FACS dot plots are shown of LSK, LT-HSC, ST-HSC and MPP cells in bone marrow. (**F**) Numbers of LSKs, LT-HSCs, ST-HSCs, MPPs, GMPs, MEPs, CMPs and CLPs in bone marrow from PBS (n = 7), DOX (n = 5) and DOX + NMN (n = 8) group mice. Data are shown as means ± standard deviations. Differences of three groups were assessed by one-way ANOVA test (**B,D,F**). ns, not significant, *$p<0.05$, **$p<0.01$, ***$p<0.001$.
